# Supplementary material for: Recurring adaptive introgression of a supergene variant that determines social organization
Source: Nat Commun. 2022 Mar 11;13:1180. doi: 10.1038/s41467-022-28806-7 (PMC8917144; doi:10.1038/s41467-022-28806-7)
Supplement: Supplementary file 3 — Description of Additional Supplementary Files [file 41467_2022_28806_MOESM3_ESM.pdf]

Description of Additional Supplementary Files

File Name: Supplementary Data 1
Description: Overview of 368 fire ant samples used, including species identities, colony identifiers, GPS coordinates, supergene variants, castes, sequencing amounts, NCBI identifiers, mapping rates to the S. invicta reference genome (samtools flagstat), RFLP results and genotype information.

File Name: Supplementary Data 2
Description: Overview of the 5,851 single-copy genes used in our analysis, including BUSCO identifiers, substitution models for coding and non-coding regions, average numbers of single nucleotide polymorphisms per individual among the 342 S. invicta, S. macdonaghi or S. richteri males, results of the maximum likelihood phylogenetic tree inference, genomic region, genomic coordinates, BUSCO profile completeness, BUSCO profile score and length.

File Name: Supplementary Data 3
Description: Pairwise Bray-Curtis dissimilarities between Sb supergene haplotypes, based on the genotypes of the 24,435 SNPs in the supergene region that were bi-allelic among the Sb samples.

File Name: Supplementary Data 4
Description: Statistics, data sources and Busco scores of fire ant assemblies used for the dating analysis.
